# Reduced Costs for *Staphylococcus aureus* Carriers Treated Prophylactically with Mupirocin and Chlorhexidine in Cardiothoracic and Orthopaedic Surgery

**DOI:** 10.1371/journal.pone.0043065

**Published:** 2012-08-14

**Authors:** Miranda M. L. van Rijen, Lonneke G. M. Bode, Diane A. Baak, Jan A. J. W. Kluytmans, Margreet C. Vos

**Affiliations:** 1 Laboratory for Microbiology and Infection Control, Amphia Hospital, Breda, The Netherlands; 2 Department of Medical Microbiology and Infectious Diseases, Erasmus University Medical Centre, Rotterdam, The Netherlands; 3 Business Information Centre, Amphia Hospital, Breda, The Netherlands; 4 Department of Medical Microbiology and Infection Control, VU University Medical Centre, Amsterdam, The Netherlands; Aligarh Muslim University, India

## Abstract

**Background:**

A multi centre double-blind randomised-controlled trial (M-RCT), carried out in the Netherlands in 2005–2007, showed that hospitalised patients with *S. aureus* nasal carriage who were treated prophylactically with mupirocin nasal ointment and chlorhexidine gluconate medicated soap (MUP-CHX), had a significantly lower risk of health-care associated *S. aureus* infections than patients receiving placebo (3.4% vs. 7.7%, RR 0.42, 95% CI 0.23–0.75). The objective of the present study was to determine whether treatment of patients undergoing elective cardiothoracic or orthopaedic surgery with MUP-CHX (screen-and-treat strategy) affected the costs of patient care.

**Methods:**

We compared hospital costs of patients undergoing cardiothoracic or orthopaedic surgery (n = 415) in one of the participating centres of the M-RCT. Data from the ‘Planning and Control’ department were used to calculate total hospital costs of the patients. Total costs were calculated including nursing days, costs of surgery, costs for laboratory and radiological tests, functional assessments and other costs. Costs for personnel, materials and overhead were also included. Mean costs in the two treatment arms were compared using the t-test for equality of means (two-tailed). Subgroup analysis was performed for cardiothoracic and orthopaedic patients.

**Results:**

An investigator-blinded analysis revealed that costs of care in the treatment arm (MUP-CHX, n = 210) were on average €1911 lower per patient than costs of care in the placebo arm (n = 205) (€8602 vs. €10513, p = 0.01). Subgroup analysis showed that MUP-CHX treated cardiothoracic patients cost €2841 less (n = 280, €9628 vs €12469, p = 0.006) and orthopaedic patients €955 less than non-treated patients (n = 135, €6097 vs €7052, p = 0.05).

**Conclusions:**

In conclusion, in patients undergoing cardiothoracic or orthopaedic surgery, screening for *S. aureus* nasal carriage and treating carriers with MUP-CHX results in a substantial reduction of hospital costs.

## Introduction

*Staphylococcus aureus (S. aureus)* nasal carriage rates range from about 20 to 50%, depending on the population and the definitions used [1]–[3]. Infections with *S. aureus* can develop after disruption of the skin barrier, for example after an incision has been made during surgery. It has been shown that in surgical patients, nosocomial *S. aureus* infections are mainly caused by their own *S. aureus* strain (endogenous infection) [4]–[7]. *S. aureus* nasal carriage is now considered to be a well-defined risk factor for subsequent infection in various groups of patients, especially those on dialysis; with cirrhosis of the liver; undergoing surgery; and with intravascular devices or in intensive care [3], [8]. This raised the hypothesis that eradication of *S. aureus* from the nose would result in fewer *S. aureus* infections in these groups of patients. Many studies have evaluated this effect in the past decades. Until 2010, only a few studies were double-blind randomised-controlled trials (RCT) [5], [9]–[16]. In these studies various patient populations were treated intranasally with mupirocin, an antibiotic nasal ointment. None of these studies found a significantly reduced number of *S. aureus* infections compared to placebo treatment. However, in most of these studies, both *S. aureus* nasal carriers and non-carriers were treated. Perl et al were the first to perform a subgroup analysis on carriers only and showed that 4.0 percent of mupirocin treated patients with nasal carriage of *S. aureus* suffered from nosocomial *S. aureus* infections, compared to 7.7 percent of those who received placebo (p = 0.02). Subsequently, all data pertaining to carriers in the above mentioned RCTs were combined in a systematic review, which showed that carriers who were treated with mupirocin before surgery had 44% less chance of developing a nosocomial *S. aureus* infections than patients receiving placebo [17].

Based on these findings a multi-centre double-blind randomised-controlled trial (M-RCT) was performed in which only *S. aureus* nasal carriers were included [6]. This study showed that patients treated with mupirocin and chlorhexidine gluconate medicated soap (MUP-CHX) had a significantly lower risk of health-care related *S. aureus* infections than patients receiving placebo (3.4% vs. 7.7%, RR 0.42, 95% CI 0.23–0.75).

The objective of the present study was to compare hospital costs of patients treated with MUP-CHX (screen-and-treat strategy) to those of patients treated with placebo (comparable to a non-screen-and-treat strategy), in patients undergoing elective cardiothoracic or orthopaedic surgery.

**Figure 1:**
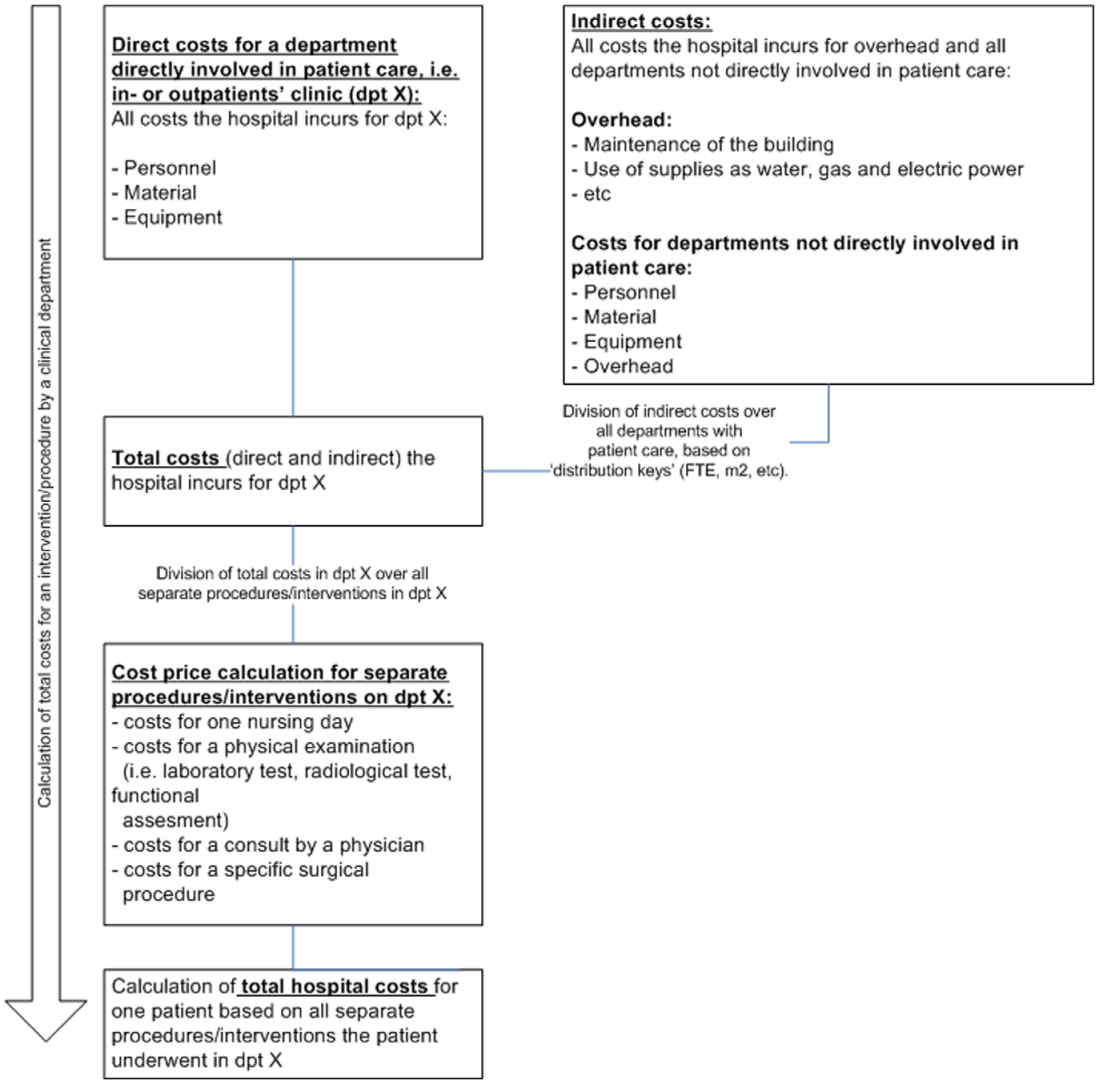
Calculation of total costs incurred by the hospital for an individual patient in a particular department.

## Methods

In the M-RCT, performed in three university hospitals and two teaching hospitals, patients who were admitted to departments of surgery and internal medicine were screened for *S. aureus* nasal carriage [6]. The present cost analysis was carried out for patients of only the Amphia hospital, a teaching hospital which serves a population of approximately 440,000 inhabitants. During the study period, on average 41,534 patients were admitted annually to this hospital with 271,528 in-patient days per year (mean number over the period 2005 to 2007, excluding day care).

**Table 1:** Mean hospital costs for patients treated with MUP-CHX or placebo.

	Mean costs per patientMup/CHX (n = 210)	Mean costs per patientPlacebo (n = 205)	p-value
**Total hospital costs**	8602.07	10513.33	**0.01**
**Total costs admission 1**	8445.94	9630.63	0.073
Costs for nursing days (excl IC) during admission 1	2867.69	3214.21	**0.023**
Costs for nursing days IC during admission 1	1472.2	2094.84	0.259
Costs for surgery during admission 1	3388.82	3496.11	0.293
Costs for laboratory tests during admission 1	301.33	333.05	0.200
Costs for radiodiagnostics and functional assessments during admission 1	66.02	83.33	0.082
Other costs (f.e. consults of physicians) during admission 1	349.97	409.09	**0.018**
**Total costs admission 2**	77.00	849.96	**0.015**
Costs for nursing days (excl IC) during admission 2	70.56	320.78	**0.029**
Costs for nursing days IC during admission 2	0	350.56	0.134
Costs for surgery during admission 2	6.24	111.02	**0.013**
Costs for laboratory tests during admission 2	0	27.2	**0.013**
Costs for radiodiagnostics and functional assessments during admission 2	0	5.5	**0.038**
Other costs (f.e. consults of physicians) during admission 2	0.2	34.9	**0.011**
**Total costs admission 3**	40.21	0.7	0.292
**Total costs for examinations and laboratory tests performed in outpatient departments during the follow-up period**	38.91	32.03	0.437

Data for cardiothoracic and orthopaedic patients are combined.

A total of 415 patients admitted for elective cardiothoracic and orthopaedic surgery in this hospital participated in the M-RCT. Cardiothoracic patients (n = 280) underwent Coronary Artery Bypass Grafting (CABG) operations with or without valve replacement (n = 88 and n = 150, respectively) or other cardiothoracic surgery (n = 3). In 39 patients the nature of cardiothoracic surgery was not further specified. Orthopaedic patients (n = 135) underwent knee replacement (n = 45), hip replacement (n = 50), spinal surgery (n = 28) or other orthopaedic procedures (n = 12) (see Table S1).

**Figure 2:**
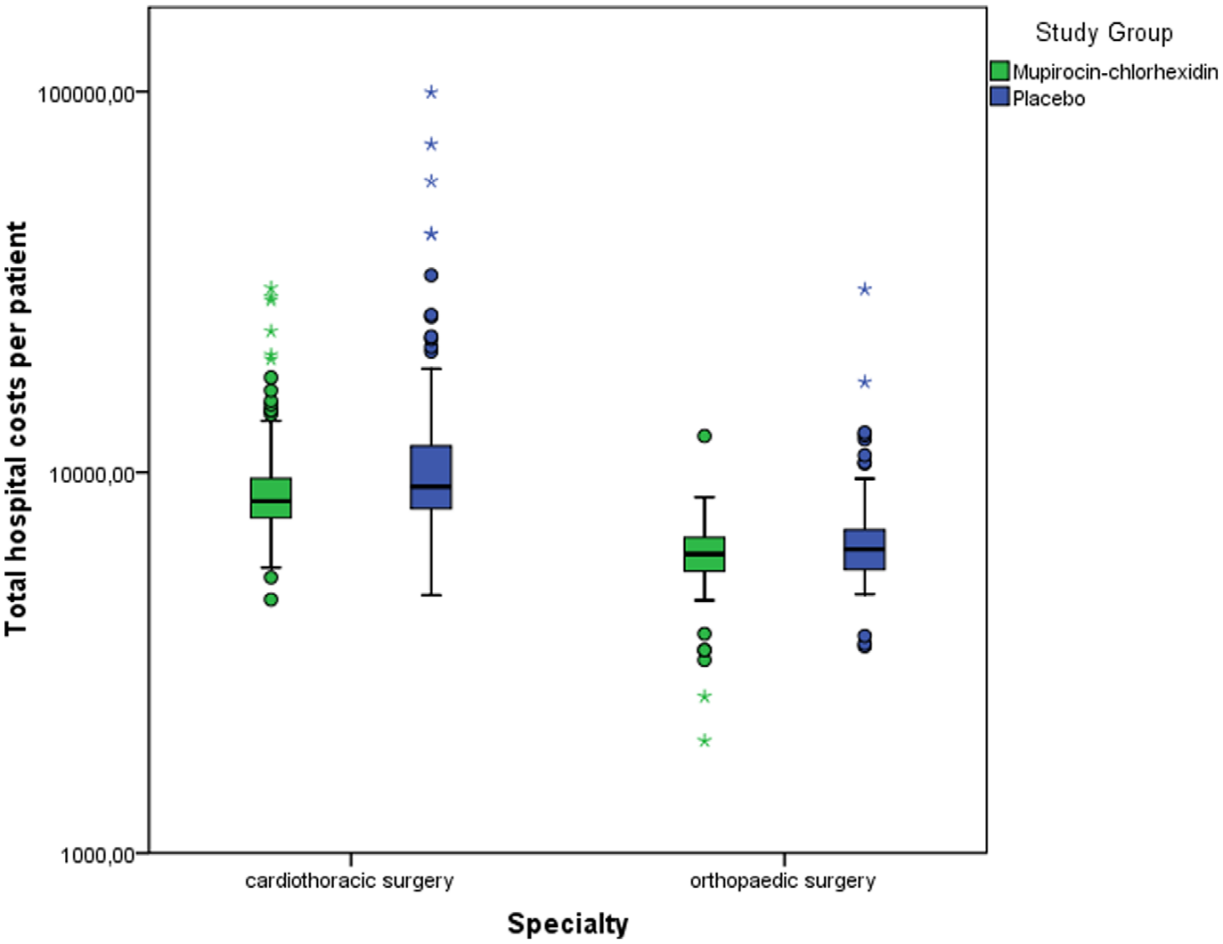
Box plot of total hospital costs for patients treated prophylactically with MUP-CHX or placebo. Total costs were estimated for the period between the dates of admission and the end of follow-up. Data are shown for cardiothoracic patients and orthopaedic surgical patients, separately. Patients with highest costs are shown in bullets • (between 1,5 and 3 times the interquartile range) and asterisks * (more than 3 times the interquartile range).

An investigator-blinded analysis was carried out to compare all hospital costs incurred between start of admission and the end of follow-up (42 days after discharge) for patients in both treatment groups (MUP-CHX vs. placebo). Costs were analysed for the total follow up period, as well as per admission (categorised as the first, second, third admission etc) during this period. Actual total hospital costs per included patient were retrieved from the data files of ‘Planning and Control’ (P&C) department of the hospital (figure 1). Since the study medication (MUP-CHX) was supplied for free during the study, the cost of this medication was added to the costs of patients treated with MUP-CHX. Screening costs were already included in the laboratory tests performed; for the placebo group, screening costs were subtracted from total costs because this study arm represents the strategy without screening or treatment. For the period between discharge and the end of follow-up, all costs made during re-admissions or costs for outpatient visits were included. Community costs were not estimated. All costs for readmissions and secondary surgical procedures in this period were included. Physicians’ fees were not registered in the P&C data file, so these costs could not be included in this analysis.

Mean costs in both treatment arms were compared using the t-test for equality of means (two-tailed). Statistical significance was accepted when p<0.05. Subgroup analysis was performed for cardiothoracic and orthopaedic patients.

The average Euro to US dollar exchange rate during the study period was 1.35.

## Results

Mean total hospital costs for a MUP-CHX treated patient undergoing cardiothoracic or orthopaedic surgery were significantly lower than costs for a placebo treated patient (€8602 vs. €10513, p = 0.01) (table 1). Table 1 shows that mean costs per patient for all individual categories, i.e. costs for nursing days, surgery, functional assessments, and laboratory and radiological tests during the first two admissions combined, were higher in the placebo group than in the MUP-CHX treated group. During the first admission significant differences between the treatment groups were found only in costs for nursing days. For the second admission, costs for nursing days, costs made during surgery, costs for laboratory and radiological tests, and functional assessments were found to be significantly lower in the treatment arm. Subgroup analysis showed that the mean expenses for MUP-CHX treated cardiothoracic patients were €2841 lower than for non treated cardiothoracic patients (€9628 vs. €12469, p = 0.006) and €955 lower for MUP-CXH treated orthopaedic patients compared to non treated orthopaedic patients (€6097 vs. €7052, p = 0.05) (figure 2).

The distribution of costs depicted in the box plot (figure 1) shows that the difference in costs between the two treatment groups is mainly caused by a number of patients with higher costs in the placebo group compared to the MUP-CHX group. This holds true for both the cardiothoracic and the orthopaedic patients. Four of these patients suffered from a deep endogenous *S. aureus* infection.

In the placebo group, 13 of 205 patients acquired a *S. aureus* infection in the hospital, compared to 3 of 210 patients in the MUP-CHX group (p = 0.01). The hospital costs for uninfected patients varied between €1986 and €72704, with a mean of €8834 and a median of €7898. For infected patients these ranged between €3693 and €99512, with a mean of €27313 and a median of €19707 (p<0.001).

## Discussion

This study shows that mean hospital costs for nasal *S. aureus* carriers undergoing elective cardiothoracic or orthopaedic surgery receiving treatment with MUP-CHX were significantly lower than for patients without treatment (placebo). This was caused by significantly higher hospital costs for *S. aureus* infected patients (p<0.001) in combination with significantly more *S. aureus* infected patients in the placebo group (p = 0.01) (see Table S1). It must be noted that for cardiothoracic surgery, nine of twenty patients with highest costs suffered from a deep *S. aureus* infection, i.e. eight cases of mediastinitis after CABG with/without valve replacement, and one case of pericarditis after pericardiectomy. Thus, almost half of the patients incurring the highest costs suffered from a deep *S. aureus* infection. This explains why prevention of these infections by application of MUP-CHX results in a significant cost reduction. In orthopaedic surgery, two deep-seated infections developed, one after total knee replacement and one after total hip revision. Costs of these two patients were found in of the group of 25 patients with highest costs.

To put these results into perspective this screen-and-treat strategy for *S. aureus* nasal carriers undergoing cardiothoracic or orthopaedic surgery would save the Amphia hospital approximately € 1,500,000 per year.

The *S. aureus* screen-and-treat strategy was already shown to result in a higher quality of patient care by reducing the number of *S. aureus* infections [6]. Lower costs and safer patient care were also found in the subgroups of patients undergoing cardiothoracic surgery or orthopaedic surgery. Other authors already estimated that introduction of a screen-and-treat strategy would result in lower hospital costs [18]–[20]. For example, the study by Wassenberg et al was based on the actual hospital costs for patients with deep-seated prosthetic joint and cardiac surgery infections in combination with the evidence-based assumptions that non-carriers have six times less chance of acquisition of such infections than *S. aureus* carriers [15], and that the relative risk of deep-seated *S. aureus* infections after MUP-CHX treatment was 0.21 compared to placebo [6]. The strength of the present study is it is the first to calculate the real hospital costs based on the data files of the P&C department. The analyses of this study were performed in an investigator-blinded fashion and patients were randomly assigned to either placebo or treatment arms [6].

The results of the present study are useful for hospitals that are planning to implement the screen-and-treat strategy but which need more evidence to convince their financial management. Some hospitals prefer to implement the treat-all strategy instead of the screen-and-treat strategy, mainly for two reasons. They argue that first, treating all patients is cheaper than screening all patients and subsequently treating nasal *S. aureus* carriers [16], [18] and second, this procedure is more convenient for the HCWs. Both a screen-and-treat and a treat-all strategy have been proven cheaper for the hospital than no screening or treatment at all. Of course, this treat-all strategy is cheaper than the screen-and-treat strategy, because the costs of the screening test, which is more expensive than the costs for mupirocin ointment and antibacterial soap, can be omitted. Wassenberg showed that treating all patients without screening would result in a saving of €7339 per life year gained, as compared to €3330 if only identified carriers were treated [18]. The low price and safety of mupirocin will easily lead to non-prudent use of this important antimicrobial agent. However, this treat-all strategy is associated with a high rate of unnecessary and thus unethical treatments that increase the likelihood of the development of resistance [21]. Mupirocin resistance will obviously lead to failure of *S. aureus* decolonisation strategies. Cautious use of mupirocin is likely to maintain the mupirocin resistance at a low level, thus preserving its efficacy. The aim of the prophylactic treatment is not to eradicate *S. aureus* forever but to result in short-term *S. aureus* eradication of approximately a month to prevent postoperative *S. aureus* wound infections. It was shown that combined low-level mupirocin and genotypic chlorhexidine resistance significantly increases the risk of persistent methicillin-resistant *S. aureus* (MRSA) carriage after decolonisation therapy [22]. Although the MRSA rates in the Netherlands are still low [23], it is useful to monitor for mupirocin and chlorhexidine resistance in hospitals using a screen-and-treat strategy for *S. aureus* carriage. In our hospital, MUP-CHX has been used for over 15 years in cardiothoracic surgery and, to date, mupirocin resistance after treatment has not been found (unpublished data).

In order to resolve practical issues, patients planned for elective cardiothoracic or orthopaedic surgery should be screened preoperatively in the outpatients department, and for those found to be a carrier, a prescription should be sent to the community pharmacy by the physician, so that patients can start treatment at home prior to admission. This treatment can be continued and finished in the hospital. For patients admitted without prior screening, rapid testing using molecular tools is an option, available 24 hours a day for optimal patient care.

The results of this study clearly show a financial benefit associated with the screen-and-treat strategy in elective cardiothoracic and orthopaedic surgery. Based on the nasal *S. aureus* carriage rate of 20% we found in the study, per thousand surgical patients approximately €400,000 could be saved. Worldwide millions of surgical procedures are performed each year, so huge numbers of patients would benefit from this strategy and this would be accompanied by large savings. The US Centers for Disease Control have now included this strategy in their top recommendations for safer health care (http://www.cdc.gov/HAI/prevent/top-cdc-recs-prevent-hai.html). For other surgical procedures or non-surgical hospitalisations, debate is still open on the economical impact of such a strategy.

## Supporting Information

Table S1 Infection data of cardiothoracic and orthopaedic patients treated with MUP-CHX or placebo.
(DOC)
