# Supplementary material for: Reduced Costs for Staphylococcus aureus Carriers Treated Prophylactically with Mupirocin and Chlorhexidine in Cardiothoracic and Orthopaedic Surgery
Source: PLoS One. 2012 Aug 14;7(8):e43065. doi: 10.1371/journal.pone.0043065 (PMC3419251; doi:10.1371/journal.pone.0043065)
Supplement: Table S1 — Infection data of cardiothoracic and orthopaedic patients treated with MUP-CHX or placebo. (DOC) [file pone.0043065.s001.doc]

| CARDIO |  |  | n | infections | endogenous | exogenous | SSI deep | SSI superficial | pneumonia |
| --- | --- | --- | --- | --- | --- | --- | --- | --- | --- |
|  | CABG | mup/chx | 83 | 1 | 0 | 1 | 0 | 0 | 1 |
|  |  | placebo | 67 | 5 | 5 | 0 | 4 | 1 | 0 |
|  | CABG + valve replacement | mup/chx | 40 | 0 | - | - | - | - | - |
|  |  | placebo | 48 | 5 | 5 | 0 | 5 | 0 | 0 |
|  | other | mup/chx | 1 | 1 | 0 | 1 | 1 | 0 | 0 |
|  |  | placebo | 2 | 0 | - | - | - | - | - |
|  | not further specified | mup/chx | 24 | 0 | - | - | - | - | - |
|  |  | placebo | 15 | 0 | - | - | - | - | - |

| ORTHO |  |  | n | infections | endogenous | exogenous | SSI deep | SSI superficial |
| --- | --- | --- | --- | --- | --- | --- | --- | --- |
|  | knee replacement | mup/chx | 19 | 0 | - | - | - | - |
|  |  | placebo | 26 | 1 | unknown | unknown | 1 | 0 |
|  | hip replacement | mup/chx | 22 | 0 | - | - | - | - |
|  |  | placebo | 28 | 0 | - | - | - | - |
|  | spine surgery | mup/chx | 13 | 0 | - | - | - | - |
|  |  | placebo | 15 | 2 | 2 | 0 | 0 | 2 |
|  | other | mup/chx | 8 | 1 | 1 | 0 | 1 | 0 |
|  |  | placebo | 4 | 0 | - | - | - | - |

CABG = coronary artery bypass grafting

SSI = surgical site infection
